# Mesenchymal stem cells versus their conditioned medium in the treatment of ischemia/reperfusion injury: Evaluation of efficacy and hepatic specific gene expression in mice

**DOI:** 10.22038/IJBMS.2022.62642.13860

**Published:** 2022-07

**Authors:** Somayeh Khosravi-Farsani, Arash Zaminy, Sedigheh Kazemi, Morteza Hashemzadeh-Chaleshtori

**Affiliations:** 1 Cellular and Molecular Research Center, Basic Health Sciences Institute, Shahrekord University of Medical Sciences, Shahrekord, Iran; 2 Department of Anatomical Sciences, School of Medicine, Shahrekord University of Medical Sciences, Shahrekord, Iran; 3 Burn and Regenerative Medicine Research Center, Guilan University of Medical Sciences, Rasht, Iran

**Keywords:** Conditioned medium, Ischemia, Liver failure, Mesenchymal stem cell, Reperfusion

## Abstract

**Objective(s):**

The mechanisms underlying the beneficial effects of MSCs on hepatic I/R injury are still poorly described, especially the changes in hepatocyte gene expression. In this study, the effect of bone marrow-derived mesenchymal stem cells (BMSCs) and adipose tissue-derived mesenchymal stem cells (AMSCs) and their conditioned medium on hepatocyte gene expression resulted by I/R shock were investigated.

**Materials and Methods:**

Liver ischemia models were induced by clamping in experimental groups. Experimental groups received MSCs or conditioned medium treatments and the control group received Dulbecco’s Modified Eagle Medium (DMEM). During 1, 24 hr, and 1 week after treatment, the serum levels of alanine aminotransferase (ALT), aspartate transaminase (AST) and lactate dehydrogenase (LDH) enzymes and tissue catalase activity (CAT) were measured. Gene expression of a number of hepatocyte-specific genes (Alb, Afp, and Ck8) and Icam-1 which is upregulated under inflammatory conditions were also evaluated in 5, 24 hr, and 1-week intervals after I/R insult.

**Results:**

In this study, liver enzymes showed a much more shift in the control group than treated groups and it was more noticeable 5 hr post-treatment. Moreover, gene expression pattern of the control group underwent changes after I/R injury. However, treated groups gene expression analysis met a steady trend after I/R insult.

**Conclusion:**

Our finding shows that stem cell treatment has better curative effects than conditioned medium. BMSCs, AMSCs or BMSC and AMSC-derived bioactive molecules injection have potential to be considered as a therapeutic approach for treating acute liver injury.

## Introduction

Ischemia-reperfusion (I/R) is recognized as a serious, worldwide surgical concern. I/R injury might grow locally or systematically, engaging multiple organs and tissues. During liver transplantation, the ischemic condition decreases membrane potential, alters the distribution of ions, and promotes metabolic acidosis by substituting aerobic patterns of metabolism for anaerobic hepatocytes and endothelial cells (1, 2). Lack of oxygen (O_2_) in ischemic conditions leads to disruption of the oxidative phosphorylation process and ultimately depletes cellular adenosine triphosphate (ATP) and creates a deleterious alteration in H^+^, Na^+^, and Ca^2+^hemostasis in hepatic cells (3). Mast cells and macrophages located at interstitial spaces near the postcapillary venules are triggered by I/R insult and elevate inflammatory responses to I/R even to remote organs. Activated Kupffer cells cause endothelial and hepatocellular injury by rapidly enhancing their release of reactive oxygen species (ROS) and pro-inflammatory cytokines such as tumor necrosis factor-alpha (TNF-α), Interleukin-1 (IL1), Interleukin-12 (IL12), and interferon-gamma (INF-γ). TNF-α activates significant expression of adhesion molecules such as intercellular adhesion molecule-1 (I-CAM-1) to facilitate endothelial cell-leukocyte adhesion (4). Hepatic injury after I/R insult is still an unresolved problem in clinical practice. Recently, the transplantation of mesenchymal stem cells (MSCs) has been identified as a therapeutic tool in different types of experimental liver injuries (5-7). Studies show that MSCs reduce oxidative stress in recipient mice and accelerate repopulation of hepatocytes after liver damage (5, 8). Previous studies have demonstrated that MSCs may play important indirect roles in liver regeneration rather than hepatic differentiation. They may provide crucial factors required for efficient healing of damaged liver (9-11). In several murine models of acute liver failure, administration of mesenchymal stem cell-conditioned medium (MSC-CM) significantly reduced liver infiltration of inflammatory cells, attenuated apoptosis, and increased proliferation of hepatocytes. As a result, it enabled liver regeneration and improved survival (2, 12-16). In most of these studies, immune cells and immunological pathways involved in acute liver failure were analyzed to manifest the healing of MSC-CM.

Previous reports demonstrated the capacity of just one kind of mesenchymal stem cell and conditioned medium in repairing the liver tissue. However, to our knowledge, the efficiency of different kinds of MSCs or MSC-secreted products in liver treatment has not been studied in the same experimental condition. This study investigates the hepatoprotective response of bone marrow-derived mesenchymal stem cells (BMSC), adipose tissue-derived mesenchymal stem cells (AMSC), conditioned medium of bone marrow stem cells (BMSC-CM), and conditioned medium of adipose mesenchymal stem cells (AMSC-CM) in an experimental model of acute liver injury and compares their effectiveness. Moreover, in this paper, the expressions of some hepatocyte-specific genes such as α-fetoprotein (*Afp*), albumin (*Alb*), and cytokeratinin8 (*Ck8*) are evaluated.

## Materials and Methods

***Animals***

7-10 weeks Balb/c male mice (body weight, 20–25 g) were housed in a temperature and humidity-controlled environment with 12 hr light and 12 hr dark cycle and free access to food (standard laboratory chow) and water. Animal care was directed in accordance with the institutional guidelines of Shahrekord University of Medical Sciences (ethics code: 3-9-92) and the National Institutes of Health (NIH) for the Care and Use of Laboratory Animals. 

***Mesenchymal stem cell isolation and culture***

Adipose tissues were removed from the inguinal region of two male mice and transferred to phosphate-buffered saline (PBS) containing 1% penicillin-streptomycin (pen/strep). The tissues were washed and then finely minced. The pieces were incubated in Dulbecco’s Modified Eagle Medium (DMEM) containing 0.5 mg/ml collagenase type IV and 1% pen/strep at 37 °C for 40 min. Furthermore, the cell suspension was centrifuged and the cell pellet was collected.

Bone marrow was isolated from the femur and tibia medullary cavity area by flushing with a 25-gauge needle using the serum-free medium (DMEM). Isolated cells were centrifuged at 1200 revolutions per minute (RPM) for 5 min.

The cells were seeded at the concentration of 5000 cells/cm^2^ and incubated at DMEM-LG (Invitrogen) supplemented with 15% fetal bovine serum (FBS), 1% pen/strep, and 1% L-glutamine solution in 37 °C and 5% CO_2_. The initial spindle-shaped cells appeared after 3 days. Cells were allowed to grow to 70–90% confluence and were passaged every 5 days with 12.5% trypsin- Ethylenediaminetetraacetic acid (EDTA). 

***Immunophenotypic analysis***

MSCs characterization was performed by flow cytometry as done previously (17, 18). At passage 3, cultured adherent cells were characterized by flow-cytometry fluorescence-activated cell sorting (FACS) Calibur equipment (Becton Dickinson) analyzing CD29, CD73, CD105, and CD90 (Peridinin chlorophyll protein complex (PerCP)-conjugated antibodies, Immunostep, Spain) as positive markers and CD45 (Fluorescein isothiocyanate (FITC)-conjugated antibody, BD Biosciences, US) as negative. Cells were exposed to a specific antibody and incubated at room temperature for 45 min, washed with PBS, and stored at 4 °C until analysis. A flow cytometer (Becton Dickinson, USA) was used to analyze the samples.

***Mesenchymal stem cell-conditioned medium (CM)***

Osugi *et al*. method (19) was conducted for mesenchymal stem cell-conditioned medium preparation with slight modifications. Briefly, 1.3×10^4^ cells/cm^2^ at passages 3–5 were allowed to grow to 70%–80% confluence in a T75 flask then washed thoroughly and cultured in 15 ml serum-free DMEM. After 48 hr, the medium was collected, centrifuged at 1200 RPM for 5 min and the supernatant was concentrated 20 times. The samples were pooled, sterilized by 0.22 µm biological filters, and stored at -70 °C. 

***Liver ischemia procedure***

Temporary warm ischemia of the liver was performed under anesthesia with ketamine 40 mg/kg and xylazine 5 mg/kg. The abdomen was opened through midline incision. The portal vein, hepatic artery, and the bile duct of the liver were clamped with a microvessel clip just above the branching to the right lateral lobe and partial liver ischemia was induced. Following clamp placement, the intestines were replaced into the abdominal cavity carefully. After 60 min of partial liver ischemia, the vessel clip was released and hepatic reperfusion was initiated. Sterile saline (500 μl) was administered to the peritoneal cavity to replace any fluid loss during surgery. In the sham group, the abdomen was opened without any clamping. Mouse abdomen was closed with 4-0 silk sutures, and animals were allowed to recover for the required reperfusion period.

***Experimental design***

The animal experiment was administrated into six groups: (I) Sham group, (II) DMEM group, (III) BMSCs group, (IV) AMSCs group, (V) BMSC-CM group, and (VI) AMSC-CM group. DMEM group was treated with DMEM; MSCs groups received mesenchymal stem cell treatment from bone marrow (BMSCs) or Adipose tissue (AMSCs). CM groups were treated with a conditioned medium of BMSCs (BMSC-CM) or AMSCs (AMSC-CM) and the sham group received no treatment. 

***Cell injection into mice***

Immediately after reperfusion onset, mice were injected intravenously via the tail vein with 200 µl DMEM in the DMEM group, 200 µl DMEM contains 1×10^6 ^BMSC or AMSCs, 200 µl CM. Animals were sacrificed after 1, 5, 24 hr, and 1 w (n=6 per each point). A liver injury test was carried out by analyzing blood and liver tissue samples which were collected and properly preserved for subsequent procedures. 

***Biochemical analysis***

To evaluate hepatocellular injury, blood samples were centrifuged for 10 min at 3000 RPM and serum samples analyzed by Autoanalyzer BT-3000 for aspartate transaminase (AST), alanine aminotransferase (ALT), and lactate dehydrogenase (LDH).

The liver catalase (CAT) activity of experimental groups in liver homogenates was performed as described previously by Aebi (20). Briefly, 995 μl H_2_O_2_ solution (composed of 10 mmol H_2_O_2_ in 50 mM phosphate buffer, pH 7.4), and 5 μl homogenate were pipetted into a cuvette. The absorbance decrease of H_2_O_2_ was followed at a wavelength of 240 nm for 2 min. Total protein samples were measured using the method of Bradford (21). CAT activity was expressed as U/mg protein.

***Gene expression analysis***

*Total RNA isolation and cDNA synthesis*

Total ribonucleic acid (RNA) was extracted from the liver tissue using the Trizol reagent (Sigma, T9424) according to the manufacturer’s instructions. The RNA pellet was dissolved in nuclease-free water and quantified at 260 nm in a NanoDrop 2000c spectrophotometer (Thermo Fisher Scientific, USA). The RNA purity was assessed by measuring the 260/280 nm absorbance ratio with appropriate purity values between 1.8 and 2.0. RNA was stored at −70 °C until further analysis. 

Five-hundred ng of total RNA was reverse transcribed using an RT reagent kit (Takara, RR037A) according to the manufacturer’s guidelines. A total of 10 μl volume reaction was conducted in a thermocycler (Gene Amp PCR System 2400, Perkin-Elmer, Boston, MA, USA). cDNA was conserved at −20 °C until used.

*Quantitative real-time -polymerase chain reaction* *(qRT-PCR)*

qRT-PCR was carried out for the candidate genes; Alpha-fetoprotein (*Afp*), Albumin (*Alb*), and Cytokeratin 8 (*Ck8*) as hepatocyte-specific genes, Intercellular Adhesion Molecule 1(*Icam*-1) as an inflammatory marker and *Gapdh* as the internal control by SYBRE Green Master Mix (Takara, RR820L) in a Corbett rotor gene 3000 real-time system. PCR primer sets were designed using the Primer3 website (http://bioinfo.ut.ee/primer3/) across two exons to avoid contamination of genomic DNA. The primers are shown in Table 1.

PCR amplification was carried out in a 10 μl reaction volume containing cDNA, SYBR Green premix, gene-specific forward and reverse primers, and DNase/RNase-free deionized water. The following cDNA amplification program was used: initial denaturation at 95 °C for 30 sec; 40 cycles of 95 °C for 10 sec, annealing (annealing temperature adapted for specific primer set used) for 15 sec, and extension at 72 °C for 20 sec. Cycling parameters were determined and analyzed using the 2^_ΔΔCT^ method. 

To determine the amplification efficiency from 10-fold serial dilutions of cDNA for each gene, standard curves were generated to get the correlation coefficients (*R*2) and slope values (22). Using these standard curves, the corresponding PCR amplification efficiencies (*E*) were calculated from the equation *E* = (10^(−1/slope)^ −1) × 100.

***Histological evaluation***

Formalin-fixed, paraffin-embedded liver samples were sectioned at 4 µm thickness and stained with hematoxylin-eosin (H&E) to observe morphological changes under a light microscope. Observations were made under an Olympus CX21 microscope. The severity of I/R injury was graded on a scale of 0-4 for sinusoidal dilatation, vacuolization of hepatocyte cytoplasm, and necrosis as previously described (23, 24). Absence of congestion, necrosis, or vacuolization is scored as 0, whereas severe congestion/ballooning, and 60% or greater lobular necrosis are given a value of 4. Five fields of tissue from each sample were assessed at 400x magnification.

***Statistical analysis***

One-way analysis of variance (ANOVA) was used for data analysis using GraphPad Prism 6.0 software. All data were expressed as mean ± standard deviation. Tukey’s *post hoc* test was carried out to indicate a statistically significant difference.

## Results

***Culture of BM-MSCs and AMSCs***

**Figure 1:**
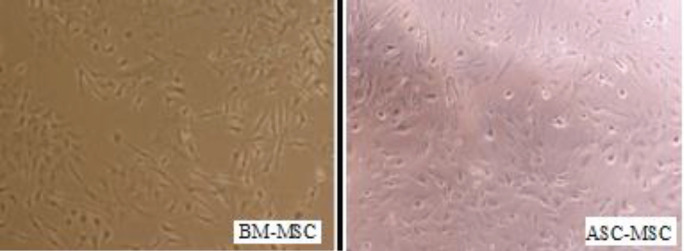
BMSCs and AMSCs morphology. Phase-contrast image showing these cells at passage 3 (4x)

**Figure 2:**
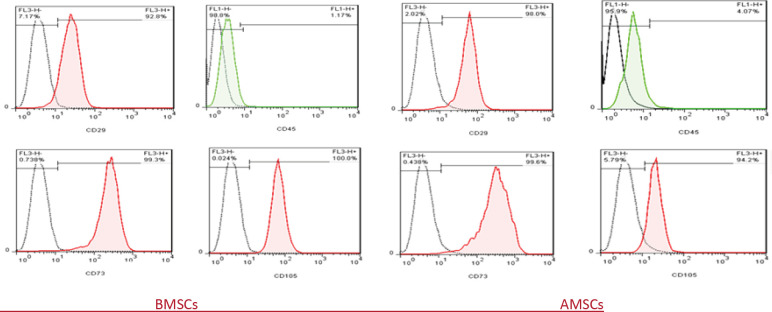
Characterization of mice-derived BMSCs and AMSCs. Cells were harvested at passage 3, labeled with the antibodies specific for mesenchymal stem cells or negative control. Flow cytometric analysis indicated that these cells were negative for CD45 but positive for MSC markers CD29, CD73, CD105, and CD90. A histogram of CD90 is shown in supplementary data (Supplementary Figure S1). These findings show that these cells displayed characteristics of BMSCs and AMSCs

BMSC: bone marrow-derived mesenchymal stem cells; AMSCs: adipose tissue-derived mesenchymal stem cells

Isolated cells from bone marrow were sub-cultured into tissue culture dishes (Figure 1). Extracted cells were washed with PBS and cultured into tissue flasks. In the first passage, cellular colonies were formed. Undesirable cells were gradually removed during the first and second passages. MSCs were spindle-shaped and adherent. In addition, we performed flow cytometric analysis. Isolated MSC showed strong CD29, CD73, CD105, and CD90 expressions (Figure 2 and supplementary Figure S1). MSC had a negative tendency to express CD45 (lymphohematopoietic stem cell marker). These results indicate that the isolated stem cells, 

***Mouse model of hepatic I/R injury***

We examined the effects of the partial occlusion of the blood flow to the hepatic lobes. Mice were awake and had free access to food and drinking water within 2 hr after surgery. No abdominal infection was detected and all of the mice survived. 

***Biochemical finding***

Liver injury was assessed by ALT, AST, LDH, and catalase (CAT) enzymes level in the serum and tissue sample. Sham-treated animals did not display noticeable alterations in the liver enzymes at each time point. The biochemical findings indicate the preventive effects of mesenchymal stem cells and their conditioned media of further hepatic injury. 

*Levels of ALT, AST and LDH in serum*

As shown in Table 2-4, the levels of AST, ALT, and LDH were higher in DMEM, MSCs, and MSCs-CM groups compared with the sham group at each time point. The serum level of ALT, AST, and LDH was abruptly elevated (*P*<0.05) in the DMEM group in the first 5 hr post-treatment. However, BMSCs, AMSCs, BMSC-CM, and AMSC-CM groups did not represent a sudden peak at 5 hr post-treatment. After 1 w, the AMSC-CM and BMSC-CM groups showed a higher level of ALT, AST, and LDH compared with the MSCs groups and there was a significant difference in LDH levels between MSCs-CM groups and the Sham group (*P*>0.05). BMSC treated group had convincingly constrained ALT, AST, and LDH levels to the lowest amount after I/R damage (Tables 2-4).

*Level of CAT in liver tissues*

DMEM group showed a remarkable decrease (*P*<0.05) in catalase activity of liver tissue after reperfusion for 1, 5 hr, and 1 w compared with those in the sham group. The catalase level was increased following MSCs and MSCs-CM treatments compared with DMEM after 1, 5 hr, and 1 w of reperfusion. After 1 hr of reperfusion, there was no significant difference in CAT levels between MSCs and MSCs-CM groups. However, a significant increase was noted in AMSC-CM compared with AMSC at 5 hr (*P*<0.05). After 1 w, AMSC-CM and BMSC-CM groups exhibited a significantly lower level of CAT compared with MSCs groups (*P*<0.05) and there were no significant differences between AMSC-CM and DMEM groups (*P*>0.05). Significant increases were seen in BMSC and AMSC groups compared with AMSC-CM and BMSC-CM groups on day 7 (Table 5).

***Gene expression***

**Figure 3:**
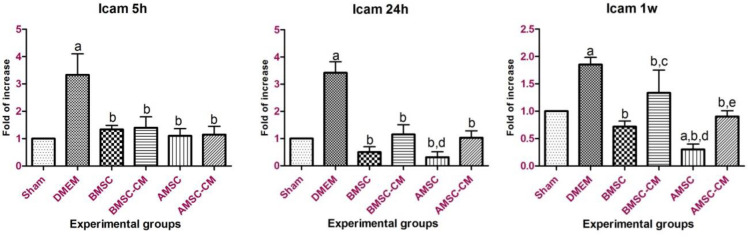
Icam-1 gene expression 5 hr, 24 hr, and 1-week post-treatment. Gene expression analysis revealed an increase in Icam mRNA expression in the DMEM group (control) along with a decrease in Icam mRNA expression after treatment by BMSCs, BMSC-CM, AMSCs, and AMSC-CM. Apparently, AMSCs treatment is more effective than other treatments (Sham group mRNA expression was considered 1). Data represented as mean ± standard deviation

**Figure 4:**
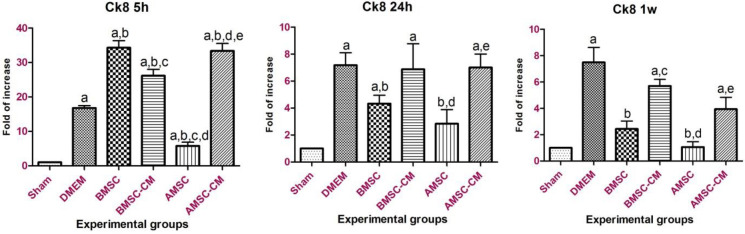
Ck8 gene expression 5 hr, 24 hr, and 1 week post-treatment. Ck8 expression faced a sudden peak 5 hr post-treatment. Yet mRNA expression of the Ck8 gene was dramatically decreased in BMSC and AMSCs groups by 24-hour till 1-week post-treatment. Data represented as mean ± standard deviation. a*P*<0.05 vs sham; b*P*<0.05 vs DMEM; c*P*<0.05 vs BMSC; d*P*<0.05 vs BMSC-CM; e*P*<0.05 vs AMSC

DMEM: dulbecco’s Modified Eagle Medium; BMSC: bone marrow-derived mesenchymal stem cells; AMSC: adipose tissue-derived mesenchymal stem cells

**Figure 5:**
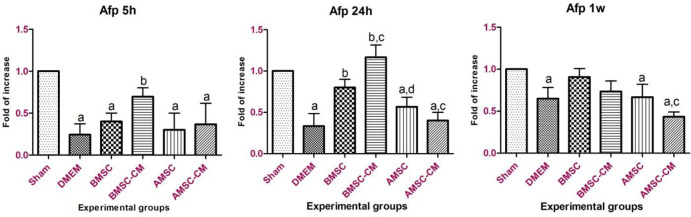
Afp gene expression 5 hr, 24 hr, and 1-week post-treatment. Afp expression was increased significantly in BMSC and BMSC-CM groups after 24 hr. Data represented as mean ± standard deviation. a*P*<0.05 vs sham; b*P*<0.05 vs DMEM; c*P*<0.05 vs BMSC; d*P*<0.05 vs BMSC-CM; e*P*<0.05 vs AMSC

DMEM: dulbecco’s Modified Eagle Medium; BMSC: bone marrow-derived mesenchymal stem cells; AMSC: adipose tissue-derived mesenchymal stem cells

The standard curves for both target and reference genes were created based on the linear relationship between CT (y-axis) versus log cDNA dilution (x-axis) (Figure S2). The corresponding amplification curve is shown in Figure S3. The amplification efficiencies listed in Table S1 indicate similar amplification efficiencies for the target and reference genes. Quantitative analysis has demonstrated that *Icam*-1- expression increased up to 24 hr post-treatment in the DMEM group as the result of inflammatory responses after I/R injury (Figure 3). On the other hand, *Icam-1 *gene overexpression in BMSC, BMSC-CM, AMSC, and AMSC-CM groups was not as significant as DMEM group which indicates the healing effect of these cells and their conditioned media on hepatocyte inflammatory responses. Surprisingly, stem cell transplantation especially AMSCs treatment was more effective than the conditioned media in the long-term healing process. In other words, conditioned media practically alleviated I/R injury in a limited timeline. *Ck8* gene expression pattern repeated this point as well and emphasized therapeutic effect of BMSCs and AMSCs after I/R injury on hepatocytes (Figure 4). *Afp* expression was up-regulated by BMSC and BMSC-CM and increased significantly after 24 hr (*P*<0.05). The expression level was more than AMSC and AMSC-CM (Figure 5). It might be needed to inject a higher amount of AMSC and AMSC-CM. Further experiments are required to investigate the cause of this event.

**Figure 6:**
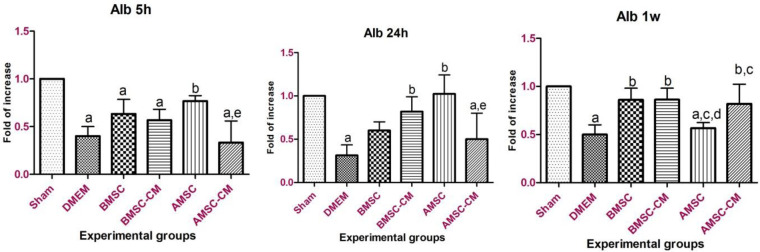
Alb gene expression 5 hr, 24 hr, and 1 week post-treatment. Alb expression was significantly decreased in the control group (*P*<0.05) and increased in treated groups. Almost all treatments proved to be effective in elevating Alb expression after I/R attack. Data represented as mean ± standard deviation. a*P*<0.05 vs sham; b*P*<0.05 vs DMEM; c*P*<0.05 vs BMSC; d*P*<0.05 vs BMSC-CM; e*P*<0.05 vs AMSC

DMEM: dulbecco’s Modified Eagle Medium; BMSC: bone marrow-derived mesenchymal stem cells; AMSC: adipose tissue-derived mesenchymal stem cells

*Alb* gene expression showed an increase in BMSCs and BMSC-CM during the time course (Figure 6). AMSCs and AMSC-CM groups faced a similar trend regarding *Alb *expression. However, AMSCs treated group decreased *Alb *expression after 1 week which entails further experiments to enlighten the cause of this event.

***Histopathological findings***

**Figure 7:**
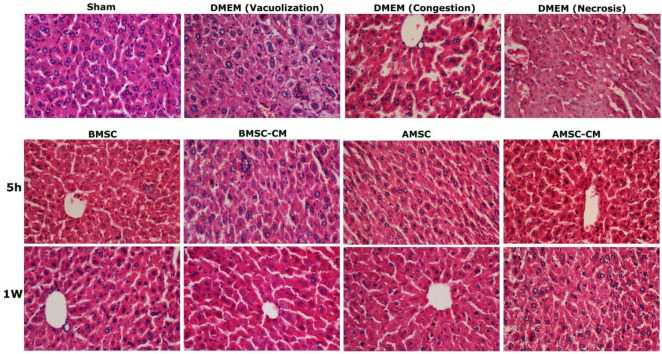
H&E staining of liver sections 5-hours and 1- week post-reperfusion in the studied groups

H&E staining was performed on tissue sections to determine the extent of sinusoidal congestion, cytoplasmic vacuolization, and necrosis (Figure 7). The severity of liver I/R injury was scored blindly using the Suzuki classification. The result displayed no sign of I/R pathology in the sham group (Table V). By contrast, Liver tissues at 5 hr and 1 w showed severe hepatic injury in the DMEM group animals (7.81±1.17 and 6.08±1.1, respectively; Figure 7 and Table 6). In addition, livers in the MSC and MSCs-CM groups displayed moderate edema and vacuolization with mild to moderate tissue necrosis at 5 hr. However, all groups displayed significantly better liver tissue pathology than the DMEM group and no significant difference was detected in the pathology of the BMSC, AMSC, BMSC-CM, and AMSC-CM groups.

Seven days after treatment by MSCs and MSCs-CM, there was a liver histology improvement that was significantly higher in the BMSC group. It can be concluded that the growth factors of the conditioned medium were cleared from the body.

**Table 1:** Specific primers for target and control genes used in RT-PCR analysis

Genes symbol	Primer sequence 5´→3´	TM (°C)
*Afp*-Forward	CTGCAAAGCTGACAACAAGGA	63
*Afp*-Reverse	CTTAATAATGGTTGTTGCCTGGAG	
*Alb*-Forward	GCCATTCTAGTTCGCTACACC	55
*Alb*-Reverse	GATTGCAGACAGATAGTCTTCCA	
*Ck8*- Forward	CAAGAAGGATGTGGACGAAGCATAC	57
*Ck8*-Reverse	AACTCACGGATCTCCTCTTCATGG	
*Icam*-1 Forward	CGTCCGCTTCCGCTACCATCA	59
*Icam*-1 Reverse	TGGCTGGCGGCTCAGTATCT	
*Gapdh*- Forward	CAGCCTCGTCCCGTAGACAA	61
*Gapdh*-Reverse	GCCGTGAGTGGAGTCATACTG	

Afp: α-fetoprotein; Alb: albumin; Ck8: cytokeratinin8; Gapdh: glyceraldehyde-3-phosphate dehydrogenase

AMSC: adipose tissue-derived mesenchymal stem cells

**Table 2:** Mouse serum levels of ALT in each group after 1, 5 hr, and 1 w reperfusion

Groups	**1 hr**	**5 hr**	**1 w **
Sham	83.33±12.58	83.33±13.02	79.33±12.33
DMEM	600±50^a^	3509±500.2^a^	280±20^a^
BMSC	203.5±20.98^a,b^	357±100.7^b^	98.67±18.72^b^
BMSC-CM	176.7±25.17^a,b^	100.7±17.79^b^	109.3±35.28^b^
AMSC	296.7±26^a,b,c,d^	500±30^b^	120±10^b^
AMSC-CM	390±30^a,b,c,d,e^	682.7±39.07^b^	142±10^a,b^

Data represented as mean ± standard deviation. a*P*<0.05 vs sham; b*P*<0.05 vs DMEM; c*P*<0.05 vs BMSC; d*P*<0.05 vs BMSC-CM; e*P*<0.05 vs AMSC

ALT: alanine aminotransferase; BMSC: bone marrow-derived mesenchymal stem cells

**Table 3:** Mouse serum levels of AST in each group after 1, 5 hr, and 1w reperfusion

Groups	**1 hr**	**5 hr**	**1 w **
Sham	119±20.05	120±18.03	123±16.09
DMEM	1033±147.4^a^	2753±484.3^a^	541.7±51.07a
BMSC	250±70^b^	350.3±100^b^	116.7±15.28^b^
BMSC-CM	620±72.11^a,b,c^	560±121.7^b^	200±40^b,c^
AMSC	300±20^a,b,d^	430±20^b^	127±21.59^b^
AMSC-CM	550±20^a,b,c,e^	620±620^b^	184±14.42^b^

Data represented as mean ± standard deviation. a*P*<0.05 vs sham; b*P*<0.05 vs DMEM; c*P*<0.05 vs BMSC; d*P*<0.05 vs BMSC-CM; e*P*<0.05 vs AMSC

AST: aspartate transaminase; DMEM: dulbecco’s Modified Eagle Medium; BMSC: bone marrow-derived mesenchymal stem cells; AMSC: adipose tissue-derived mesenchymal stem cells

**Table 4:** Mouse serum levels of LDH in each group after 1, 5 hr, and 1w reperfusion

Groups	**1 h**	**5 h**	**1 w **
Sham	455±62.65	434±65.02	451.3±70.04
DMEM	2483±525.2^a^	6683±852^a^	5124±372.5^a^
BMSC	1150±199.7^b^	2651±804.6^a,b^	714.3±85.01^b^
BMSC-CM	950±132.3^b^	3334±585.5^a,b^	826.7±75.06^a,b^
AMSC	1533±351.2^a,b^	2808±952.8^a,b^	724.3±75.01^b^
AMSC-CM	1057±125^b^	2878±651.8^a,b^	920±43.59^a,b^

Data represented as mean ± standard deviation. a*P*<0.05 vs sham; b*P*<0.05 vs DMEM; c*P*<0.05 vs BMSC; d*P*<0.05 vs BMSC-CM; e*P*<0.05 vs AMSC

LDH: lactate dehydrogenase; DMEM: dulbecco’s Modified Eagle Medium; BMSC: bone marrow-derived mesenchymal stem cells; AMSC: adipose tissue-derived mesenchymal stem cells

**Table 5:** Level of CAT (catalase) activity in rat liver tissues in each group after 1, 5 hr, and 1 w reperfusion

Groups	**1 hr**	**5 hr**	**1 w **
Sham	124.7±2.51	113.2±2.09	112.2±1.11
DMEM	67.79±5.52^a^	59.04±3.09^a^	71.81±3.15^a^
BMSC	91.53±7.06^a,b^	83.12±2.06^a,b^	91.22±4.52^a,b^
BMSC-CM	86.24±3.84^a,b^	79.83±2.37^a,b^	81.49±3.16^a,b,c^
AMSC	83.70±4.9^a,b^	67.79±2.33^a,b,c,d^	101±2.66^a,b,c,d^
AMSC-CM	95.93±6.54^a,b^	86.53±5.46^a,b,e^	75.22±5.11^a,c,e^

Data represented as mean ± standard deviation. a*P*<0.05 vs sham; b*P*<0.05 vs DMEM; c*P*<0.05 vs BMSC; d*P*<0.05 vs BMSC-CM; e*P*<0.05 vs AMSC

DMEM: dulbecco’s Modified Eagle Medium; BMSC: bone marrow-derived mesenchymal stem cells; AMSC: adipose tissue-derived mesenchymal stem cells

a*P*<0.05 vs sham; b*P*<0.05 vs DMEM; c*P*<0.05 vs BMSC; d*P*<0.05 vs BMSC-CM; e*P*<0.05 vs AMSC

DMEM: dulbecco’s Modified Eagle Medium; BMSC: bone marrow-derived mesenchymal stem cells; AMSC: adipose tissue-derived mesenchymal stem cells

**Table 6:** Suzuki Score: The level of sinusoidal congestion, vacuolization and necrosis in the experimental groups

Groups	**5 hr**	**1 w **
Sham	0	0
DMEM	7.81±1.17^a^	6.08±1.1^a^
BMSC	3.36±1.03^a,b^	1.94±0.55^a,b^
BMSC-CM	4.5±0.87^a,b^	4±0.83^a,b^^,^^c^
AMSC	3.73±1.28^a,b^	2.15±0.48^a,b,c^
AMSC-CM	4.96±1.01^a,b^	3.63±0.63^a,b^

a*P*<0.05 vs sham; b*P*<0.05 vs DMEM; c*P*<0.05 vs BMSC; d*P*<0.05 vs BMSC-CM; e*P*<0.05 vs AMSC. Data are mean ± standard deviation

BMSC: bone marrow-derived mesenchymal stem cells; AMSC: adipose tissue-derived mesenchymal stem cells

## Discussion

Depriving blood supply during organ transplantation has been realized as a major post-surgery complication. For instance, in the United States of America 1.3 million people are affected by ischæmic injury each year (25). The immunomodulatory effect of MSCs has attracted the focus of researchers to investigate their significant immunosuppression of both innate and adaptive immune systems (26). MSCs are also known for their tissue healing effect, especially in acute kidney injury (AKI), acute myocardial infarction (AMI), stroke, and liver and lung injury (13, 27-29). Previous studies on MSC transplantation to liver tissue indicated their differentiation capacity to hepatocytes and protective effect of MSCs against major insults induced by inflammatory responses and oxidative stress in a rodent model of hepatic I/R (7, 30). To the best of our knowledge, this report is the first to describe the healing effect of BMSCs and AMSCs themselves along with their conditioned medium.

As a case in point, H&E staining of injured tissue displayed recovery of BMSC, BMSC-CM, AMSC, and AMSC-CM treated mice after one week. It was consistent with the biochemical findings in ALT, AST, LDH, and CAT levels, while DMEM-treated mice demonstrated dramatic changes such as congestion, vacuolization, and necrosis in hepatic tissue. 

The increased serum levels of ALT, AST, and LDH, and decreased level of tissue catalase activity at 5 hr post-treatment demonstrate the hepatocellular damage after injury. In comparison with the other time points, hepatic injury after reperfusion for 5 hr was more severe which is consistent with the previous study (31). These liver index figures were reduced 24 hr after treatment, which shows the ameliorated state of hepatic tissue after I/R injury. Furthermore, treatments by MSCS (AMSC and BMSC) and MSCs-CM (BMSC-CM and AMSC-CM) significantly improved the levels of ALT, AST, LDH, and CAT in comparison with the DMEM group. In this regard, there was no appreciable difference between MSCs and MSCs-CM after 5 hr, although both AMSC and BMSC demonstrated higher efficacy after one week of reperfusion. As a result, it appears that the cell transplantation groups have a therapeutic advantage over the CM groups in the liver failure model mice in the long term.

*Icam*-1 is expressed at a low concentration in the liver and other tissues; however, *Icam-*1 mRNA level is readily up-regulated by inflammatory cytokines (32). As mentioned previously, the production of TNF-α as the result of activated Kupffer cells induces *Icam*-1 gene expression in the sinusoidal endothelial cells. Then, ICAM-1 facilitates neutrophil infiltration and leads to hepatocellular dysfunction and tissue injury (4). MSCs produce paracrine-targeted micro-vesicles containing bioactive molecules that suppress inflammatory responses (30). Relative analysis of gene expression represented a comparable recovery of hepatocytes in MSCs and MSCs-CM groups by maintaining *Icam*-1 expression at low level 5, 24 h, and 1 w post-treatment which was induced by I/R injury. These findings further support the idea that MSC transplantation to damaged tissues is associated with cell adhesion and proliferation, preventing apoptosis and endothelial cell-leukocyte attachment, and expanding chemokines contributed to tissue repair (30, 33).

In the current study, the expression of some hepatocyte-specific genes such as cytokeratin 8 (marker of hepatocytes), α-fetoprotein (hepatoblast marker), and albumin (typical marker of mature hepatocytes) was evaluated and compared in different experimental groups.

It was shown that the expression of these markers is up-regulated in MSCs treated with injured liver tissue under *in vitro* conditions (34, 35). Gene expression analysis of liver tissue at the mRNA level showed that *Ck8* has over-expressed in almost all groups of study at 5 and 24 hr after reperfusion. This may be due to MSCs differentiation or the influence of paracrine factors on hepatogenic differentiation in injured liver tissues (34). In contrast, after 7 days, the *Ck8* expression levels in liver tissue from BMSC and AMSC groups decreased significantly compared with BMSC-CM, AMSC-CM groups, and DMEM group. A number of studies have shown that *Ck8* plays essential roles in protecting hepatocytes from stress (36, 37). Therefore, the increase in *Ck8* level can be associated with persistent injury in these groups. On the other hand, enhanced *Afp* expression following treatment with BMSCs, AMSCs, and their conditioned medium substantially declared the effective impact of BMSCs and AMSCs and their conditioned medium injection on the maintenance of hepatocyte gene expression. *Alb* gene expression was alleviated by inducing reperfusion following 60 min of ischemia in the control group. This finding is in agreement with Alrefaei’s findings which declared that *Alb* expression diminished after I/R injury (38). However, treatments with BMSCs, AMSCs, and their conditioned medium triggered *Alb* expression, and pattern of *Alb* gene expression faced a steady rise from 5 hr to 1 week post-treatment. It should be noted that the conditioned medium of both BMSCs and AMSCs was beneficial for short-term recovery which can be due to the limited number of trophic factors and bioactive molecules in the conditioned medium. Long-term recovery of hepatic tissue was executed by stem cells; a relatively permanent source of anti-inflammatory chemokines, and proliferative and regenerative factors for retrieval of damaged hepatocytes.

Taken together, the findings of this study confirmed previous reports on the impact of stem cell transplantation on hepatic recovery after I/R injury (39-41). All treatments by stem cells and their conditioned medium have proven to be fruitful. According to gene expression patterns and enzyme levels, BMSC-CM seemed to be more effective than AMSC-CM suggesting more dosage of AMSC-CM should be administered in order to be effective. Further studies with more focus on the long-term healing effect and different doses of conditioned medium on hepatic tissue are therefore recommended.

## Conclusion

It was found that BMSCs and AMSCs or their conditioned medium injection after I/R injury would protect the liver organ from break down and restore hepatocytes’ function to an acceptable level. Hepatic enzymes, gene expression, and histological evaluations indicated that our treatments had beneficial effects on the liver. Moreover, CM can be considered a possible treatment agent in hepatic IR injury. Since this study does not contain information about the different doses of conditioned medium treatment on hepatic IR injury, further experimental and clinical studies are needed. 

## Authors’ Contributions

MH Devised the project and supervised the work. SKh Designed the study. SKh and SK Carried out the experiments. AZ Performed the flow cytometry analysis. SKh and AZ Wrote the manuscript in consultation with MH and SK.

## Funding Sources

This study was financially supported by Shahrekord University of Medical Sciences, Iran. [Grant Number 1530].

## Supplementary Material

Characterization of mice-derived BMSCs and AMSCs for MSC marker CD90 was carried out and flow cytometric analysis indicated that these cells were positive for MSC marker CD90. Moreover, the standard and amplification curves at 10-fold serial dilutions of cDNA were created. Then, the amplification efficiencies for all genes were calculated which showed similar amplification efficiencies for the target and reference genes.

## Conflicts of Interest

None declared.
